# Associations of urinary sodium levels with overweight and central obesity in a population with a sodium intake

**DOI:** 10.1186/s40795-018-0255-6

**Published:** 2018-11-21

**Authors:** Juyeon Lee, Yunji Hwang, Kyoung-Nam Kim, Choonghyun Ahn, Ho Kyung Sung, Kwang-Pil Ko, Kook-Hwan Oh, Curie Ahn, Young Joo Park, Suhnggwon Kim, Young-Khi Lim, Sue K. Park

**Affiliations:** 1 Department of Preventive Medicine, College of Medicine, Seoul National University, 103 Daehakro, Seoul, Jongnogu 03080 South Korea; 2 Department of Biomedical Science, College of Medicine, Seoul National University, 103 Daehakro, Seoul, Jongnogu 03080 South Korea; 3 Cancer Research Institute, Seoul National University, 103 Daehakro, Seoul, Jongnogu 03080 South Korea; 4 Division of Public Health and Preventive Medicine, Seoul National University Hospital, 101 Daehakro, Seoul, Jongnogu 03080 South Korea; 5 Department of Preventive Medicine, Gachon University College of Medicine, 38-13 Dokjeom-ro 3beon-gil, Incheon, Namdong-gu South Korea; 6 Division of Nephrology, Department of Internal Medicine, Seoul National University Hospital, 101 Daehakro, Seoul, Jongnogu 03080 South Korea; 7 Division of Endocrinology, Department of Internal Medicine, Seoul National University Hospital, 101 Daehakro, Seoul, Jongnogu 03080 South Korea; 8 Seoul K-Clinic, 18-5, Changgyeonggung-ro 34-gil, Seoul, Jongnogu 03077 South Korea; 9 Department of Radiological Science, Gachon University College of Medicine, 191 Hambangmoe-ro, Incheon, Yeonsu-gu South Korea

**Keywords:** Sodium excretion, Obesity, Body mass index, Waist circumference, Korean National Health and nutrition examination survey

## Abstract

**Background:**

Previous studies have reported an association between dietary sodium intake and overweight/central obesity. However, dietary survey methods were prone to underestimate sodium intake. Therefore, this study investigated the associations of calculated 24-h urinary sodium excretion, an index of dietary sodium intake, with various obesity parameters including body mass index (BMI) and waist circumference (WC) in a population with a relatively high sodium intake.

**Methods:**

A total of 16,250 adults (aged ≥19 years) and 1476 adolescents (aged 10-18 years), with available information on spot urine sodium levels and anthropometric measurements from the Korea National Health and Nutrition Examination Survey (KNHANES) were included in this study. We calculated 24-h urine sodium excretion levels from spot urine sodium levels using the Tanaka formula.

**Results:**

In adults, those with high sodium excretion levels (≥ 3200 mg) showed increased odds of overweight and central obesity compared to those with low urinary sodium excretion level (< 2200 mg) (odds ratio [OR] = 2.17, 95% confidence interval [CI] = 1.90-2.49 for overweight; OR = 2.50, 95% CI = 2.13-2.94 for central obesity). These associations were also observed in adolescents (OR = 5.80, 95% CI = 3.17-10.60 for overweight; OR = 4.19, 95% CI = 1.78-9.89 for central obesity).

**Conclusions:**

The present study suggests that reducing salt intake might be important for preventing overweight and central obesity, especially in adolescents. However, because the present study was conducted with cross-sectional study design, further longitudinal studies are warranted to confirm the causal relationship between urinary sodium excretion and overweight/central obesity.

**Electronic supplementary material:**

The online version of this article (10.1186/s40795-018-0255-6) contains supplementary material, which is available to authorized users.

## Background

Overweight, which is commonly defined as body mass index (BMI) ≥ 25 kg/m^2^ on World Health Organization [WHO] guideline [1], is a critical concern to global public health [2]. It has been associated with numerous chronic diseases, such as cardiovascular disease, chronic kidney disease, type 2 diabetes mellitus, hypertension, and hyperlipidemia [3, 4]. While BMI is a useful measure to define stages of obesity, it does not reflect the body composition and fat distribution of an individual. Therefore, other anthropometric measures such as waist circumference (WC), a measure for central obesity, coupled with BMI may better identify obesity-related health risks [5].

In Korea, about 30% of the adult population are overweight (including obese) and about 4% are obese [6]. Contributing to this rise in obesity, the increase in Westernized diet containing fattening and high-sodium westernized foods (hot dogs, fries, and cheese sandwiches) in addition to traditional Korean salty, pickled foods (Kimchi and salted seafood) have been noted as one of the main risk factors. Dietary intake has been noted dietary components [7]. It is important to detect risk factors related overweight and central obesity that use effective intervention.

On average, the Korean population consumes 4847 mg sodium per day which is over double the amount of the WHO recommended value of 2000 mg/day [8]. Given the high intake of sodium and rising rates of obesity among Koreans, it is critical to investigate the association between dietary sodium intake and obesity.

Previous studies have reported an association between sodium intake and overweight /obesity in Korea [9, 10]. However, sodium intake was estimated by using inaccurate methods such as dietary recall and food frequency questionnaire [11–13] which are prone to underestimation of sodium intake [14]. Because most people usually tend to report healthier eating habits than their true eating habits [15]. The urinary sodium excretion level is the most reliable method to evaluate dietary sodium intake [16, 17].

Therefore, in this study, we examined the associations of calculated 24-h urinary sodium excretion levels, a marker for dietary sodium intake, with overweight and central obesity in Korea. We also evaluated whether urinary sodium excretion level is associated with certain types of obesity, such as only overweight without central obesity, only central obesity without overweight, or overweight combined with central obesity, relative to normal BMI and WC. In addition, because it was suggested that overweight and central obesity risk by sodium intake is larger in adolescents than adults [18], we investigated the associations of calculated 24-h urinary sodium excretion levels with overweight and central obesity among different age groups.

## Methods

### Study population

The Korea National Health and Nutrition Examination Survey (KNHANES) is a population-representative cross-sectional survey based on complex, stratified, and multistage cluster sampling [19]. Details of KNHANES have been published elsewhere [20]. In brief, this survey was designed to collect information on the health and nutrition of all individuals resided in the Republic of Korea. The survey is conducted by the Korea Centers for Disease Control and Prevention (KCDC). Interviews were based on a standardized questionnaire, asking information regarding health behaviors, past histories, and dietary intake. Blood and urine laboratory tests were conducted according to standardized protocols by well-trained medical experts, and equipment was calibrated periodically. Dietary sodium intake and nutrient consumption was estimated by 24-h recall and food questionnaire from a nutritional survey conducted by a well-trained nutritionist. The dietary intake of food consumptions were assessed using a validated semi-quantitative FFQ developed for KNHANES [21]. The food consumption questionnaire has been designed as an open-ended survey. Study subjects were asked to estimate the average amounts of serving of 109 food items and the frequencies of consumption in last 12 months. Daily nutrient intakes were calculated by combining serving average of serving, portion per unit and frequency per day for each food item. Nutritional assessment was analyzed using CAN-Pro 4.0 (Korean Nutrition Society, Seoul, Korea).

**Fig. 1:**
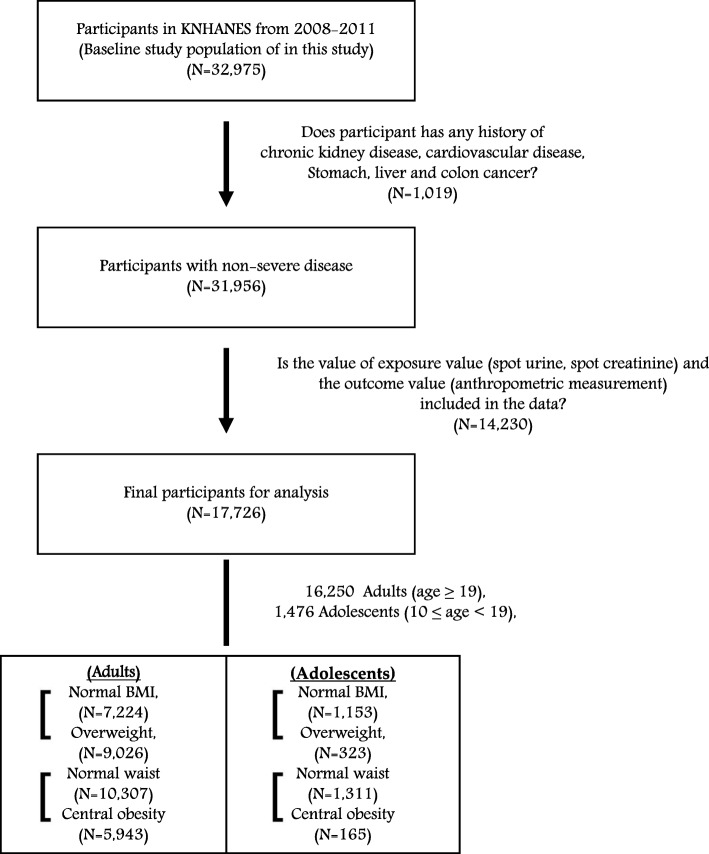
Study subjects to assess the association between calculated 24-h urinary sodium excretion level and obesity, the Korea National Health Examination and Nutritional Survey (KNHANES) Phase IV-V, 2008-2011

In total, 32,975 individuals who participated in the 4th and 5th KNHANES in 2008-2009 and 2010-2011 were included as the eligible population. Of them, we excluded those with a past history of chronic kidney disease, cardiovascular disease, stomach cancer, liver cancer, colon cancer at enrollment (*N* = 1019), because these patients were more likely to experience anorexia, resulting in poor nutrient intake habits. We further excluded 14,230 participants with missing information on exposure variables (spot urine sodium and spot urine creatinine) and the outcome variables, including anthropometric measurements (height, weight, or WC). Therefore, the final study population consisted of 17,726 participants, including 16,250 adults (age ≥ 19 years) and 1476 adolescents (aged 10–18 years) (Fig. 1). The age groups definition for adults and adolescents are used to World Health Organization (WHO) guidelines [22]. This study was conducted according to the guidelines established by the Declaration of Helsinki. All participants aged 19 years or older gave written informed consents for inclusion before they participated in the study. In participants aged less than 19 years, written informed consents were obtained from their parents or guardians on behalf of the study participants, after both study participants and their parents or guardians read and understand the process of the study. Also, research staff checked whether each informed consent form was completed and signed by the participants aged less than 19 years as well as parent or guardian. Study protocols were approved by the institutional review board (IRB) of Seoul National University Hospital (IRB No: 1612-083-814).

### Anthropometric measurements and defined of overweight and central obesity

The anthropometric measurements (height, weight, and WC) were measured in the mobile examination center following a standardized measurement protocol by trained KNHANES staff [19]. Height was measured to the nearest 0.1 cm using a Seca 225 (Germany Seca Ltd.), and weight was measured to the nearest 0.1 kg with a GL-6000-20 (Korea G-tech Ltd.). BMI (kg/m^2^) was calculated using the height and weight data according to the following equation: [BMI = weight (kg)/height^2^ (m^2^)]. WC was measured at the narrowest area between the lowest rib and the uppermost lateral border of the right iliac crest and the measured to the nearest 1 mm with a Seca 200 (Germany Seca Ltd.).

The World Health Organization (WHO) defined an adolescent as any person between ages 10 and 19 [22]. In adults and adolescents, overweight was defined as BMI ≥ 23 kg/m^2^, based on Asian BMI guideline proposed by the WHO Western Pacific Region in 2000 [23].

In adults, central obesity was defined as WC ≥ 90 cm in men and ≥ 80 cm in women, based on the modified National Cholesterol Education Program Adult Treatment Panel (NECP ATP- III) cut-offs in Asians [24]. In adolescents, central obesity was defined as age- and sex- specific WC ≥ 90th percentile, using the criterion of the International Diabetes Federation [25].

### Assessment of exposure: Calculated 24-h urinary sodium excretion

Urinary sodium levels were measured using the Hitachi 7600 ISE special reagent (Japan Hitachi Ltd.). Urinary creatinine concentrations were calculated based on a colorimetric method (Hitachi Automatic Analyzer, Hitachi, Japan). Urinary sodium and creatinine were measured in the same central laboratory for all participants. The KNHANES provided urine creatinine and sodium values from the overnight spot urine samples, and did not provide information on the 24-h urinary sodium excretion level [26]. Although the measurement of 24-h urine sodium excretion level is considered to be the most reliable method to estimate the dietary sodium intake [16, 27–30], collecting 24-h urinary sodium is difficult in a large population study and can be inaccurate because of participants skipping some urine collection throughout the day [16, 27–29]. In the present study, we estimated calculated 24-h urinary sodium excretion levels using the following Tanaka’s formulas (Eqs 1, 2) [31].

1 $$ {\displaystyle \begin{array}{l}24\hbox{-} \mathrm{h}\ \mathrm{urinary}\ \mathrm{creatinine}\ \mathrm{excretion}\ \left(\mathrm{mg}\right)=\hbox{-} 2.04\times \mathrm{age}\ \left(\mathrm{year}\right)+14.89\times \mathrm{weight}\ \left(\mathrm{kg}\right)+16.14\times \\ {}\mathrm{height}\ \left(\mathrm{cm}\right)\hbox{-} 2244.45.\end{array}} $$

2 $$ {\displaystyle \begin{array}{l}\mathrm{Calculated}\ 24\hbox{-} \mathrm{h}\ \mathrm{urinary}\ \mathrm{sodium}\ \mathrm{excretion}\ \left(\mathrm{mg}\right)=21.98\times {\mathrm{XNA}}^{0.392}.\\ {}\mathrm{XNA}=12\times 24\hbox{-} \mathrm{h}\kern0.5em \mathrm{urinary}\ \mathrm{creatinine}\ \mathrm{excretion}\ \left(\mathrm{mg}\right)\end{array}} $$

The calculated 24-h urinary sodium excretion levels were then classified into three categories (< 2200 mg [< 100 mEq], 2200–3199 mg [100-139 mEq], and ≥ 3200 mg [≥ 139 mEq]). In the present study population, the lowest sodium level (< 2200 mg [< 100 mEq]) [32] is close to roughly 6 g of a daily salt intake, which is recommended guidelines in previous studies [33].

### Covariates

Sociodemographic factors such as age (continuous), sex (male/female), monthly household income (KRW), education, marital status were assessed. Monthly household income (KRW) was categorized as < 1,500,000, 1,500,000-2,999,999, 3,000,000-3,999,999, 4,000,000 or above. Education status was categorized as below middle school, high school, college, university or above. Marital status was categorized into married or single, others (including never married, separated, divorced or bereaved). Hypertension (no/yes) was defined as a person with anti-hypertensive medication or systolic BP ≥ 140 mmHg, diastolic BP ≥ 90 mmHg, or the presence of a medical history of hypertension. Diabetes mellitus (no/yes) was defined as fasting blood glucose ≥126 mg/mL or the presence of a history of diabetes. Other covariate included lifestyle factors such as drinking, smoking, and dietary intake of water, energy, potassium (continuous). Drinking status was categorized as no, yes (including ever-drinkers and current drinkers). Smoking status was categorized into no, yes (including ex-smokers and current smokers). Physical activity (no/yes) was defined as performing regular exercise enough to sweat once a week or more.

### Statistical analysis

We followed the KNHANES data analysis guidelines provided by the KCDC [34]. We conducted analysis of variance tests for continuous variables and chi-square tests for categorical variables to evaluate the difference in the distribution or count of selected characteristics among three groups. We evaluated the associations between calculated 24-h urinary sodium excretion levels and overweight and central obesity using polytomous logistic regression models. This study was adjusted for the clinical importance confounders, considered in previous study and study participants demographic and clinical characteristics: age (continuous), sex (male/female), diet energy intake (mg/day), water intake (g/day), potassium intake (mg/day), and physical activity (no/yes) (Table 1). We examined multi-collinearity between independent variables with Pearson correlation coefficient and Variance inflation factor.

**Table 1:** General characteristics of study subjects, Korea National Health Examination and Nutritional Survey (KNHANES), Phase IV-V, 2008-2011

	Urinary sodium excretion levels (mg/day)			
	< 2300	2300-3199	3200 ≤	*P*-vaule^b^
Total population [*N* = 17,726]	*N* (%)	*N* (%)	*N* (%)	
Age (years) ^a^				
≥ 19	1316 (8.1)	6261 (38.5)	8673 (53.4)	< 0.01
10 – 18	308 (20.9)	734 (49.7)	434 (29.4)	
Sub-population				
Adults [*N* = 16,250]				
Sex				
Male	502 (7.2)	2646 (38.1)	3788 (54.6)	< 0.01
Female	814 (8.7)	3615 (38.8)	4885 (52.4)	
Monthly household income (KRW)				
< 1,500,000	252 (7.6)	1148 (34.7)	1911 (57.7)	< 0.01
1,500,000–2,999,999	335 (8.3)	1499 (37.4)	2178 (54.3)	
3,000,000–3,999,999	352 (8.0)	1750 (39.6)	2320 (52.4)	
≥ 4,000,000	363 (8.4)	1801 (41.5)	2178 (50.2)	
Education status				
Below Middle school	613 (7.0)	3014 (34.6)	5087 (58.4)	< 0.01
High school	238 (7.8)	1243 (40.7)	1570 (51.5)	
College	282 (10.6)	1187 (44.7)	1188 (44.7)	
Highr than University	178 (10.0)	789 (44.4)	809 (45.5)	
Marital status				
Married	980 (6.9)	5287 (37.3)	7923 (55.8)	< 0.01
Single	333 (16.4)	965 (47.2)	737 (36.2)	
Others	3 (15.8)	8 (42.1)	8 (42.1)	
Drinking				
No	153 (6.5)	823 (35.0)	1377 (58.2)	< 0.01
Yes	1150 (8.3)	5391 (39.2)	7224 (52.5)	
Smoking				
No	771 (8.1)	3676 (38.6)	5064 (53.2)	0.91
Yes	531 (8.0)	2534 (38.4)	3532 (53.5)	
Physical activity				
No	877 (8.3)	4056 (38.4)	5627 (53.3)	0.23
Yes	380 (7.8)	1941 (39.7)	2570 (52.5)	
Hypertension				
No	931 (8.6)	4415 (40.9)	5447 (50.5)	< 0.01
Yes	385 (7.1)	1846 (33.8)	3226 (59.1)	
Diabetes mellitus				
No	1138 (8.2)	5481 (39.6)	7234 (52.2)	< 0.01
Yes	178 (7.4)	780 (32.5)	1439 (60.0)	
	Mean ± SD	Mean ± SD	Mean ± SD	
Diet Water intake (g/day)	972.8 ± 681.4	1002.4 ± 663.4	1010.3 ± 670.2	0.32
Diet Energy intake (kcal/day)	1894.4 ± 920.3	1968.1 ± 869.5	2007.4 ± 854.5	< 0.01
Diet Potassium intake (mg/day)	2910.1 ± 1567.2	3050.0 ± 1565.0	3180.3 ± 1584.1	< 0.01
Adolescents [*N* = 1476]				
Sex				
Male	147 (17.7)	394 (47.3)	288 (34.7)	< 0.01
Female	161 (24.9)	340 (52.5)	146 (22.6)	
Physical activity				
No	83 (20.7)	198 (49.4)	120 (29.9)	0.19
Yes	97 (16.8)	281 (48.8)	198 (24.4)	
Hypertension				
No	308 (21.0)	731 (49.9)	426 (29.1)	< 0.01
Yes	0 (0)	3 (27.3)	8 (72.7)	
Diabetes mellitus				
No	283 (21.1)	667 (49.7)	391 (29.2)	0.70
Yes	25 (18.5)	67 (49.6)	43 (31.8)	
	Mean ± SD	Mean ± SD	Mean ± SD	
Diet Water intake (g/day)	854.6 ± 497.4	858.9 ± 464.1	941.8 ± 547.6	0.01
Diet Energy intake (kcal/day)	2123.3 ± 892.8	2109.4 ± 797.9	2392.2 ± 965.4	< 0.01
Diet Potassium intake (mg/day)	2542.6 ± 1385.7	2586.9 ± 1198.4	2966.9 ± 1436.9	< 0.01

^a^Adults 19 years and older; Adolescents between 10 and 18 years

^b^For continuous variable, the ANOVA test was used. For categorical variables, the chi-square test was used

To visualize the correlation among dietary sodium intake, 24-h urinary sodium excretion, and obesity, contour plots were drawn by the ‘lattice’ package in R version 3.2.2 (http://www.r-project.org). Other statistical analyses were conducted using the SAS version 9.4. (SAS Institute Inc., Cary, NC, U.S.A).

## Results

The general characteristics of the participants are presented in Table 1. The participants included 16,250 adults (age ≥ 19 years) with an average age of 50.2 years, and 1476 adolescents (aged 10–18 years) with an average age of 13.5 years. In adults, compared with the participants low sodium excretion (< 2200 mg), those with high sodium excretion (≥ 3200 mg) were more likely to be male, drinker and smokers who exercised more, were married, had lower education levels (below middle school), had a monthly household income lower than 1,500,000 KRW), higher diet water, energy, potassium intake, presence of hypertension and diabetes mellitus. In adolescents, compared with the participants low sodium excretion, those with high sodium excretion were more likely to be female, presence of hypertension, higher diet water, energy, potassium intake.

Table 2 present the associations of calculated 24-h urinary sodium excretion levels with overweight (classified by BMI) and central obesity (classified by WC). We found increased odds of overweight and central obesity in the participants with high urinary sodium excretion (≥ 3200 mg) compared to participants in low urinary sodium excretion (< 2200 mg) (OR = 2.17, 95% CI = 1.90-2.49 in adults; OR = 5.80, 95% CI = 3.17-10.60 in adolescents for overweight; OR = 2.50, 95% CI = 2.13-2.94 in adults; OR = 4.19, 95% CI = 1.78-9.89 in adolescents for central obesity). Higher urinary sodium excretion levels were associated with increased risk of BMI and WC in different age groups, especially in adolescents.

**Table 2:** Adjusted odds ratios (ORs) for overweight and central obesity by urinary sodium excretion levels among adults and adolescents, the Korea National Health Examination and Nutritional Survey (KNHANES) Phase IV-V, 2008-2011

	Overweight classified by BMI ^c^				Central obesity classified by waist^d^			
Urinary sodium excretion levels^a^(mg/day)	Normal weight^c^	Overweight^c^			Normal waist^4^	Central obesity^d^		
	*N* (%)	*N* (%)	OR (95% CI)^2^	*P* ^e^	*N* (%)	*N* (%)	OR (95% CI)^b^	*P* ^e^
Adults	[*N* = 7224]	[*N* = 9026]			[N = 10,307]	[*N* = 5943]		
< 2200	783 (10.8)	533 (5.9)	1.00		1017 (9.9)	299 (5.0)	1.00	
2200-3199	3120 (43.2)	3141 (34.8)	1.48 (1.29-1.69)	0.03	4415 (42.8)	1846 (31.1)	1.48 (1.26-1.74)	0.02
≥ 3200	3321 (46.0)	5352 (59.3)	2.17 (1.90-2.49)	< 0.01	4875 (47.3)	3798 (63.9)	2.50 (2.13-2.94)	< 0.01
Adolescents	[*N* = 1153]	[*N* = 323]			[*N* = 1311]	[*N* = 165]		
< 2200	283 (24.5)	25 (7.7)	1.00		299 (22.8)	9 (5.4)	1.00	
2200-3199	621 (53.9)	113 (35.0)	1.47 (0.82-2.65)	0.89	676 (51.6)	58 (35.1)	1.09 (0.49-2.44)	0.67
≥ 3200	249 (21.6)	185 (57.3)	5.80 (3.17-10.60)	< 0.01	336 (25.6)	98 (59.4)	4.19 (1.78-9.89)	< 0.01

^a^24h-urinary sodium excretion levels estimated by Tanaka equation

^b^Adjusted for age and sex, energy intake (per day), water intake (per day), potassium intake (per day), and physical activity

^c^Body mass index (BMI) in adults and adolescents were classified to two groups using the steering Committee of the Regional Office for the Western Pacific Region of WHO, the International Association for the Study of Obesity and the International Obesity Task Force proposed the appropriateness of the classification of obesity in Asia in 2000 [19]. Normal weight (< 23.0 kg/m^2^), and overweight (≥ 23.0 kg/m^2^)

^d^Waist circumference (WC) in adults were classified two groups using the criterion of NECP ATP- III guideline [21], such as normal waist (male: < 90 cm, female: < 80 cm), central obesity (male: ≥ 90 cm, female: ≥ 80 cm); WC in adolescents was classified two groups using the criterion of International Diabetes Federation (IDF) [22]

^e^*P*-value

Table 3 shows the association of different types of obesity by calculated 24-h urinary sodium excretion levels in adults and adolescents. The types of obesity were classified as ‘normal BMI and normal WC’, ‘overweight without central obesity’, ‘central obesity without overweight’, and ‘overweight combined with central obesity’. The adults in the highest urinary sodium category (≥ 3200 mg) exhibited increased odds of ‘overweight without central obesity’ (OR = 1.63, 95% CI =1.35-1.98), ‘central obesity without overweight’ (OR = 2.36, 95% CI = 1.41-3.94), and ‘overweight combined with central obesity’ (OR = 2.95, 95% CI = 2.49-3.49) compared with the adults in the lowest urinary sodium category (< 2200 mg). The associations were consistent in adolescents (OR = 6.90, 95% CI = 3.25-14.63 for overweight without central obesity; OR = 5.28, 95% CI = 2.24-12.40 for overweight combined with central obesity). As shown in Additional file 1: Table S1 and S2, when we conducted sensitivity analyses using the sodium intake estimated from spot urine sodium levels, the results were robust and similar to the results using the Tanaka equation.

**Table 3:** Adjusted odds ratios (ORs) for different types of obesity^a^ by urinary sodium excretion levels in adults and adolescents, Korea National Health Examination and Nutritional Survey (KNHANES), Phase IV-V, 2008-2011

Urinary sodium excretion levels^b^ (mg/day)	Normal BMI & Normal WC	Only Overweight^c^ Without Central obesity^d^			Only Central obesity^d^ Without Overweight^c^			Overweight^c^ combined With Central obesity^d^		
	*N* (%)	*N* (%)	OR (95% CI)^e^	*P* ^f^	*N* (%)	OR (95% CI)^e^	P^f^	*N* (%)	OR (95% CI)^e^	*P* ^f^
Adults^c^	[*N* = 6722]	[*N* = 3585]			[*N* = 502]			[*N* = 5441]		
< 2200	757 (11.3)	260 (7.2)	1.00		26 (5.2)	1.00		273 (5.0)	1.00	
2200-3199	2952 (43.9)	1463 (40.8)	1.37 (1.14-1.65)	0.02	168 (33.5)	1.43 (0.85-2.40)	0.06	1678 (30.8)	1.63 (1.35-1.98)	0.03
≥ 3200	3013 (44.8)	1862 (51.9)	1.63 (1.35-1.98)	< 0.01	308 (61.3)	2.36 (1.41-3.94)	< 0.01	3490 (53.1)	2.95 (2.49-3.49)	< 0.01
Adolescents^c^	[*N* = 1140]	[*N* = 171]			[*N* = 13]			[*N* = 152]		
< 2200	283 (24.8)	16 (9.4)	1.00		0 (0)	1.00		9 (5.9)	1.00	
2200-3199	613 (53.8)	63 (36.8)	1.97 (0.79-2.40)	0.26	8 (61.5)	N/A	N/A	50 (32.9)	1.11 (0.49-2.51)	0.09
≥ 3200	244 (21.4)	92 (53.8)	6.90 (3.25-14.63)	< 0.01	5 (38.5)	N/A	N/A	93 (61.2)	5.28 (2.24-12.40)	< 0.01

^a^ Adiposity status in all populations were classified into four groups, including normal WC and normal BMI, Only overweight without central obesity, only central obesity without overweight and overweight combined with central obesity

^b^ 24 h-urinary sodium excretion levels estimated by Tanaka equation

^c^ Overweight in adults and adolescents was defined as BMI ≥ 23.0 kg/m^2^ based on Asian BMI guideline proposed by the WHO Western Pacific Region [22]

^d^ Central obesity in adults was defined as WC ≥ 90 cm in men and ≥ 80 cm in women, based on the criterion of modified NECP ATP- III guideline in Asians [24]; the central obesity in adolescents was defined as sex-specific WC ≥ 90 percentile based on the criterion of International Diabetes Federation (IDF) [25]

^e^ Adjusted for age and sex, energy intake (per day), water intake (per day), potassium intake (per day), and physical activity

^f^*P*-value

Table 4 presents the adjusted odds ratios for overweight and central obesity by dietary sodium to total calorie (Na/Kcal) ratio and dietary sodium to potassium (Na/K) ratio among adults and adolescents. The participants in the highest tertiles of Na/Kcal ratio exhibited increased odds of being classified as overweight and central obesity relative to normal BMI and WC compared with participants in the lowest tertiles of Na/Kcal ratio (OR = 1.16, 95% CI = 1.03-1.31 in adults; OR = 1.93, 95% CI = 1.27-2.95 in adolescents for overweight; OR = 1.12, 95% CI = 0.97-1.29 in adults; OR = 1.27, 95% CI = 0.73-2.21 in adolescents for central obesity). The participants in the highest tertiles of Na/K ratio exhibited increased odds of being classified as overweight and central obesity relative to normal BMI and WC compared with participants in the lowest tertiles of Na/K ratio (OR = 1.07, 95% CI = 0.94-1.21 in adults; OR = 1.64, 95% CI = 1.05-2.57 in adolescents for overweight; OR = 1.12, 95% CI = 0.98-1.29 in adults; OR = 1.58, 95% CI = 0.80-3.12 in adolescents for central obesity). This study was observed more stronger and consistent association Na and overweight, central obesity in adolescents.

**Table 4:** Adjusted odds ratios (ORs) for overweight and central obesity by dietary sodium to potassium ratio and sodium to total calorie ratio among adults and adolescents, Korea National Health Examination and Nutritional Survey (KNHANES), Phase IV-V, 2008-2011

Dietary Na/Calorie ratio^b^	Overweight^c^		Central obesity^d^		Dietary Na/K ratio^b^	Overweight^c^		Central obesity^d^	
	OR (95% CI)	P^e^	OR (95% CI)	P^e^		OR (95% CI)	P^e^	OR (95% CI)	P^e^
Adults					Adults				
1 T (< 1.90)	1.00		1.00		1 T (< 1.29)	1.00		1.00	
2 T (1.90-2.80)	1.10 (0.97-1.24)	0.49	1.17 (1.02-1.35)	0.04	2 T (1.29-1.86)	0.95 (0.84-1.08)	0.22	1.07 (0.93-1.22)	0.80
3 T (≥ 2.80)	1.16 (1.03-1.31)	< 0.01	1.12 (0.97-1.29)	0.49	3 T (≥ 1.87)	1.07 (0.94-1.21)	0.06	1.12 (0.98-1.29)	0.08
Adolescents					Adolescents				
1 T (< 1.48)	1.00		1.00		1 T (< 1.27)	1.00		1.00	
2 T (1.48-2.10)	1.31 (0.83-2.06)	0.74	0.73 (0.38-1.41)	0.13	2 T (1.27-1.75)	1.46 (0.90-2.35)	0.50	1.34 (0.67-2.67)	0.24
3 T (≥ 2.10)	1.93 (1.27-2.95)	< 0.01	1.27 (0.73-2.21)	0.10	3 T (≥ 1.75)	1.64 (1.05-2.57)	0.04	1.58 (0.80-3.12)	0.82

^a^ 24 h-urinary sodium excretion levels estimated by Tanaka equation

^b^ Adjusted for age and sex, energy intake (per day), water intake (per day), potassium intake (per day), and physical activity

^c^ Body mass index (BMI) in adults and adolescents were classified to two groups using the steering Committee of the Regional Office for the Western Pacific Region of WHO, the International Association for the Study of Obesity and the International Obesity Task Force proposed the appropriateness of the classification of obesity in Asia in 2000 [19]. Normal weight (< 23.0 kg/m^2^), and overweight (≥ 23.0 kg/m^2^);

^d^ Waist circumference (WC) in adults were classified two groups using the criterion of NECP ATP- III guideline [21], such as normal waist (male: < 90 cm, female: < 80 cm), abdominal obesity (male: ≥ 90 cm, female: ≥ 80 cm); WC in adolescents was classified two groups using the criterion of International Diabetes Federation (IDF) [22]

^e^ P-value

**Fig. 2:**
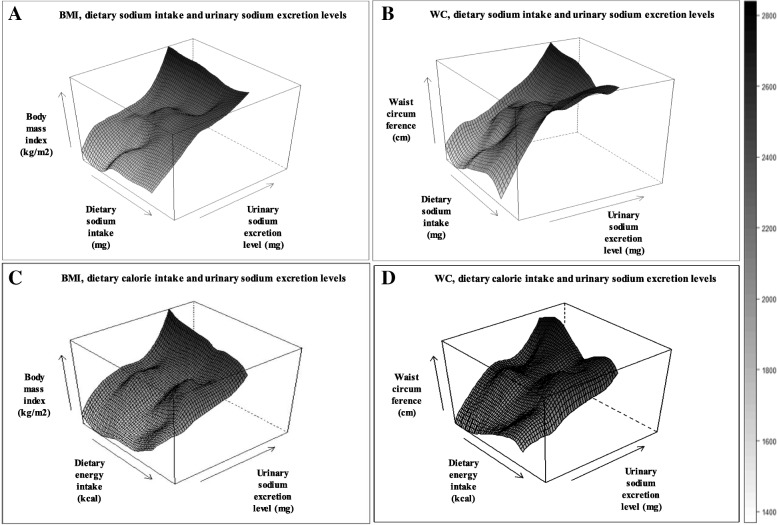
Visualization of the interplay among BMI, WC, dietary sodium intake and dietary energy intake on urinary sodium excretion level in total population. **a** BMI, dietary sodium intake (mg/day), and urinary sodium excretion levels (mg/day), (**b**) WC, dietary sodium intake (mg/day), and urinary sodium excretion levels (mg/day), (**c**) BMI, dietary calorie intake (mg/day), and urinary sodium excretion levels (mg/day), (**d**) WC, dietary calorie intake (mg/day) and urinary sodium excretion. Black color means higher value and grey lower value

We illustrated the correlations among dietary sodium intake, 24-h urinary sodium excretion, BMI, and WC in Fig. 2. In these 3-dimensinal figures, the dietary sodium intake was positively correlated with a urinary sodium excretion levels and the increase in both variables was correlated with an increase in BMI and WC, despite a fluctuating pattern was observed in its association with dietary sodium intake (Fig. 2a & b). The slope appears to be abrupt in the WC; whereas energy intake and urinary sodium excretion were also positively correlated, with an increase in BMI (Fig. 2c & d).

## Discussions

In the present study, higher urinary sodium excretion levels were associated with an increased likelihood of overweight and central obesity measured by both BMI and WC than among those with low sodium excretion. These associations were more pronounced in the adolescents compared to those in the adults.

Our results are consistent with previous studies. A few cross-sectional studies directly investigated the association between urinary sodium excretion levels and overweight and obesity in adults [12], adolescents [35]. Other cross-sectional studies reported the associations between high urine sodium excretion levels and increased body fat levels compared with participants with low levels of urine sodium excretion [12, 36]. Similarly, a longitudinal US adult population study reported that individuals with high sodium intake had increased BMI, WC, and predictive body fatness [11]. In a representative sample of the adult Spanish population, urinary sodium excretion was found to be associated with obesity. Specifically, participants with high sodium intake had overall greater energy intake and unhealthy lifestyles which were associated with the increased risk for being overweight and central obesity [37].

On the other hand, other studies reported no association between sodium intake and being overweight/central obesity. A longitudinal population study reported that Danish with higher 24-h urinary sodium excretion were not associated with changes in BMI and WC [36]. These inconsistencies in results may be due to varying sodium sensitivity among populations. It has been reported that sodium sensitive people tend to weight more than sodium-resistant people [38, 39]. Other possible reason for discrepancies maybe from differing population age, gender, clinical conditions and racial background [40, 41]. Thus, it is important to consider population sample characteristics carefully when interpreting study results.

In the present study, the stronger associations between calculated 24-h urinary sodium excretion levels and overweight/central obesity were observed in adolescents than adults. Given the limited number of studies focusing solely on adolescent’s sodium excretion and their overweight/obesity status, we could not compare our results. However, similar studies could provide some explanations. The Dortmund Nutritional and Anthropometric Longitudinally Designed study showed that the intake of processed salty food could have a negative effect on body weight in adolescents [35].

Another cross-sectional study reported that white and African-American, high sodium intake was associated with adiposity and inflammation independent of sugar-sweetened beverage and total energy intake among adolescents [7]. Dietary habit preferences of adolescents are greatly influenced by their parents [42, 43]. Parents exhibiting diets with high sodium intake had a greater chance of high energy intake due to higher fat and/or protein intake, and overall unhealthy life style choices [44], which can lead to obesity. Given the relevance of this issue to adolescents, we suggest that proper dietary sodium control targeting for families, including both parents and children, is necessary. Recent study reported that persistent obesity during adolescents and children was significantly associated with decreased estimated glomerular filtration (eGFR) [45]. This is why they have to change their eating habits since childhood.

While the exact mechanism for sodium intake and weight gain is unclear, several biological mechanisms have been proposed. Biologically, higher levels of sodium in the body can lead to being overweight by increasing cortisol levels and its metabolites [46], decreasing adiponectin, increasing anemia risk, and inducing abnormal metabolic profiles, such as high blood pressure, insulin resistance, increased triglyceride levels, and reduced HDL-cholesterol levels [46, 47]. Another possible mechanism was that high sodium intake increased the volume of extracellular water that caused increase weight gain [48]. A genetic predisposition that affects hyper-sodium intake was suggested as a plausible mechanism in the association between sodium intake and overweight [47]. In mice, those fed a higher dietary sodium intake had associated with leptin resistance and obesity. This activated the aldose reductase-fructokinase pathway in the liver stimulating endogenous fructose production and metabolism [49]. More studies are needed to confirm these effects in humans.

Several limitations should be considered when interpreting our results. First, our results cannot confirm the casual relationship between calculated 24-h urinary sodium excretion and overweight and central obesity, because the data were obtained from a cross-sectional study design. To minimize possibilities of reverse causality, we excluded participants with chronic kidney disease, chronic liver disease, cardiovascular disease, stomach cancer, liver cancer, and colon cancer that potentially resulted in altered dietary habits and uremic malnutrition after diagnosis [50], affecting their sodium levels. Malnutrition is a usual finding in patients with chronic diseases, and has been reported to affect various body compositions such as loss of lean body mass, adipose tissue, and visceral proteins. Protein loss particularly includes skeletal muscle, and impaired immune system. Protein kinetics are decided in patients with uremic on conservative treatment by amino acid, the results direct that the rates of protein synthesis and degradation are decreased whole body and skeletal muscle levels [51, 52]. Second, we used simple equations of calculated 24-h urinary sodium excretion from spot urine specimens. Because it is not feasible to measure 24-h urinary sodium excretion we used the Tanaka’s equations to estimate calculated 24-h urinary sodium excretion. A number of different equations for estimating 24-h urinary sodium excretion are currently in use. Presently, the Tanaka’s equation is widely used in clinical practice in Korea [53–55]. Although, some bias by overestimating low sodium intake ranges, underestimating high sodium intake ranges and high sensitivity were observed for the Tanaka’s equations among Tanaka, Cockcroft-Gault, a newly derived Korea equation [56, 57]. Third, controlled urinary potassium excretion levels are recommended when using urinary sodium excretion. However, this study did not test for urinary potassium excretion. To address this limitation, we adjusted the dietary potassium intake. Fourth, Unhealthy lifestyle are potentially associated with obese. Processed foods tend to be nutritionally imbalanced, added sodium, sugar, and chemical additives and have a highly energy contents [58]. Due to the present study’s cross-sectional design, we overcome the gap in eat different dietary sodium intake between low-end participants (normal individuals) and high-end participants (obese individuals). This result showed that the participants in the higher Na/Kcal ratio increased odds of being overweight relative to normal BMI (Table 4). It cannot be fully explained by overweight and obese itself a driver for excessive urinary sodium excretion. Nevertheless, it is observed that higher Na/Kcal ratio is related to overweight, so not all of the results are caused by reverse causation. So need to reduce both dietary sodium and total calorie intake are recommended (Fig. 2). Finally, we did not adjust our data for subjects undergoing pubertal stage. The pubertal stage in adolescents require more information than the biological age, and urinary sodium excretion may be different due to active steroidogenesis in puberty [59].

Despite these limitations, the present study has important strengths. First, the present study used a well-designed survey data (the KNHANES) representative of Korean population. The KNHANES collected high-quality data using standardized protocols. Second, we used more objective urinary sodium data. Urinary sodium excretion levels reflect more objective measurement for sodium intake compared with other methods [16, 27–29]. Third, a relatively large sample size allowed us to stratify participants into numerous subgroups with sufficient statistical power, potentially providing important evidence that adverse health effects of high-salt consumptions can be more enhanced in early ages. Finally, we used comprehensive information related to demographic, lifestyles, and health conditions, and these factors were assessed as covariates in the analytical models.

## Conclusions

In summary, higher urinary sodium excretion levels were associated with an increased likelihood of overweight and central obesity in adults, and adolescents. The results of the present study suggest that sodium intakes need to be reduced particularly in adolescents, to prevent overweight and central obesity and subsequent chronic diseases, including type 2 diabetes, hypertension, and cardiovascular diseases. Guidelines recommending reduction of sodium intake and school interventions for the management of obesity associated with sodium intake in adolescents should be considered. However, owing to the cross-sectional design, we cannot establish the causality between sodium intake and overweight and central obesity. Further studies, especially with longitudinal study design are warranted to confirm the results.

## Additional file

Additional file 1: **Table S1**. Adjusted for odds ratios (ORs) for overweight and central obesity by spot urine sodium levels among adults and adolescents, the Korea National Health Examination and Nutritional Survey (KNHANES) Phase IV-V, 2008-2011. **Table S2**. Adjusted for odds ratios (ORs) for different types of obesity by spot urine sodium levels in adults and adolescent, the Korea National Health Examination and Nutritional Survey (KNHANES) Phase IV-V, 2008-2011. (DOCX 34 kb)

## Abbreviations

BMI: Body mass index
CI: Confidence intervals
KNHANES: The Korean National Health and Nutrition Examination Survey
NECP ATP: the National Cholesterol Education Program Adult Treatment Panel
OR: Odds ratios
WC: Waist circumference
