# Supplementary material for: Associations of urinary sodium levels with overweight and central obesity in a population with a sodium intake
Source: BMC Nutr. 2018 Nov 21;4:47. doi: 10.1186/s40795-018-0255-6 (PMC7050808; doi:10.1186/s40795-018-0255-6)
Supplement: Supplementary file 1 — Table S1. Adjusted for odds ratios (ORs) for overweight and central obesity by spot urine sodium levels among adults and adolescents, the Korea National Health Examination and Nutritional Survey (KNHANES) Phase IV-V, 2008-2011. Table S2. Adjusted for odds ratios (ORs) for different types of obesity by spot urine sodium levels in adults and adolescent, the Korea National Health Examination and Nutritional Survey (KNHANES) Phase IV-V, 2008-2011. (DOCX 34 kb) [file 40795_2018_255_MOESM1_ESM.docx]

**Table S1.** Adjusted odds ratios (ORs) for overweight and central obesity by spot urine sodium levels among adults and adolescents, the Korea National Health Examination and Nutritional Survey (KNHANES) Phase IV-V, 2008-2011

|  | **Over weight classified by BMI** ^3^ | | | | **Central obesity classified by waist**^4^ | | | |
| --- | --- | --- | --- | --- | --- | --- | --- | --- |
| **The spot urine sodium level (mg/day)** | **Normal weight^3^** | **Overweight^3^** | | | **Normal waist^4^** | **Central obesity^4^** | | |
|  | N (%) | N (%) | OR (95% CI)^2^ | *P*^5^ | N (%) | N (%) | OR (95% CI)^2^ | *P* ^5^ |
| **Adults** | **[N=7,224]** | **[N=9,026]** |  |  | **[N=10,307]** | **[N=5,943]** |  |  |
| < 2200 | 2,221 (30.7) | 2,288 (25.3) | 1.00 |  | 2,990 (29.0) | 1,519 (25.6) | 1.00 |  |
| 2200-3199 | 2,268 (31.4) | 2,941 (32.6) | 1.20 (1.09-1.33) | 0.02 | 3,265 (31.7) | 1,944 (32.7) | 1.19 (1.08-1.31) | 0.03 |
| ≥ 3200 | 2,735 (37.9) | 3,979 (42.1) | 1.30 (1.18-1.44) | <0.01 | 4,052 (39.3) | 2,480 (41.7) | 1.30 (1.17-1.44) | <0.01 |
| **Adolescents** | **[N=1,153]** | **[N=323]** |  |  | **[N=1,311]** | **[N=165]** |  |  |
| < 2200 | 358 (31.0) | 70 (21.7) | 1.00 |  | 393 (30.0) | 35 (21.2) | 1.00 |  |
| 2200-3199 | 340 (29.5) | 92 (28.5) | 1.47 (0.83-2.51) | 0.89 | 386 (29.4) | 46 (27.9) | 1.03 (0.52-2.08) | 0.51 |
| ≥ 3200 | 455 (39.5) | 161 (49.8) | 2.08 (1.30-3.33) | <0.01 | 532 (40.6) | 84 (50.9) | 1.58 (0.87-2.88) | 0.08 |

^1^ Sodium intake estimated from spot urine samples.

^2^ Adjusted for age and sex, energy intake (per day), water intake (per day), potassium intake (per day), and physical activity.

^3^ Body mass index (BMI) in adults and adolescents were classified to two groups using the steering Committee of the Regional Office for the Western Pacific Region of WHO, the International Association for the Study of Obesity and the International Obesity Task Force proposed the appropriateness of the classification of obesity in Asia in 2000 [19]. Normal weight (< 23.0kg/m^2^), and overweight (≥ 23.0 kg/m^2^);

^4^ Waist circumference (WC) in adults were classified two groups using the criterion of NECP ATP- III guideline [21], such as normal waist (male: < 90 cm, female: < 80 cm), central obesity (male: ≥ 90 cm, female: ≥ 80 cm); WC in adolescents was classified two groups using the criterion of International Diabetes Federation (IDF) [22].

^5^ P-value.

**Table S2.** Adjusted odds ratios (ORs) for different types of obesity by spot urine sodium levels in adults and adolescents, Korea National Health Examination and Nutritional Survey (KNHANES), Phase IV-V, 2008-2011

| **The spot urine sodium level (mg/day)** | **Normal BMI & Normal WC** | **Only Overweight^2^**  **Without Central obesity^3^** | | | **Only Central obesity^3^**  **Without Overweight^2^** | | | **Overweight^2^ combined**  **With Central obesity^3^** | | |
| --- | --- | --- | --- | --- | --- | --- | --- | --- | --- | --- |
|  | N (%) | N (%) | OR (95% CI)^5^ | P^6^ | N (%) | OR (95% CI)^5^ | P^6^ | N (%) | OR (95% CI)^5^ | P^6^ |
| **Adults^3^** | **[N=6,722]** | **[N=3,585]** |  |  | **[N=502]** |  |  | **[N=5,441]** |  |  |
| < 2200 | 2,083 (31.0) | 907 (25.3) | 1.00 |  | 138 (27.5) | 1.00 |  | 1,381 (25.4) | 1.00 |  |
| 2200-3199 | 2,108 (31.4) | 1,157 (32.3) | 1.14 (0.99-1.31) | 0.59 | 160 (31.9) | 1.08 (0.81-1.44) | 0.21 | 1,784 (32.8) | 1.25 (1.12-1.40) | 0.02 |
| ≥ 3200 | 2,531 (37.6) | 1,521 (42.3) | 1.22 (1.07-1.39) | 0.02 | 204 (40.6) | 1.29 (0.98-1.70) | 0.08 | 2,276 (41.8) | 1.39 (1.24-1.56) | <0.01 |
| **Adolescents^3^** | **[N=1,140]** | **[N=171]** |  |  | **[N=13]** |  |  | **[N=152]** |  |  |
| < 2200 | 353 (31.0) | 40 (23.4) | 1.00 |  | 5 (38.5) | 1.00 |  | 30 (19.7) | 1.00 |  |
| 2200-3199 | 338 (29.6) | 48 (28.1) | 1.58 (0.37-3.07) | 0.75 | 2 (15.4) | 1.09 (0.37-2.99) | 0.69 | 44 (28.9) | 1.27 (0.63-2.57) | 0.85 |
| ≥ 3200 | 449 (39.4) | 83 (48.5) | 2.09 (1.24-3.51) | 0.01 | 6 (46.1) | 1.11 (0.23-1.19) | 0.09 | 78 (51.3) | 2.04 (1.06-3.94) | 0.03 |

^1^ Sodium intake estimated from spot urine samples.

^2^ Overweight in adults and adolescents was defined as BMI ≥ 23.0 kg/m^2^ based on Asian BMI guideline proposed by the WHO Western Pacific Region [22].

^3^ Central obesity in adults was defined as WC ≥ 90 cm in men and ≥ 80 cm in women, based on the criterion of modified NECP ATP- III guideline in Asians [24]; the central obesity in adolescents was defined as sex-specific WC ≥ 90 percentile based on the criterion of International Diabetes Federation (IDF) [25].

^4^ Adjusted for age and sex, energy intake (per day), water intake (per day), potassium intake (per day), and physical activity.

^5^ Adiposity status in all populations were classified into four groups, including normal WC and normal BMI, Only overweight without central obesity, only central obesity without overweight and overweight combined with central obesity.

^6^ P-value.
